# Investigating the antifibrotic effect of the antiparasitic drug Praziquantel in in vitro and in vivo preclinical models

**DOI:** 10.1038/s41598-020-67514-4

**Published:** 2020-06-30

**Authors:** Justin Komguep Nono, Kai Fu, Thabo Mpotje, Georgianna Varrone, Nada Abdel Aziz, Paballo Mosala, Lerato Hlaka, Severin Donald Kamdem, Daigen Xu, Thomas Spangenberg, Frank Brombacher

**Affiliations:** 1 Division of Immunology and South African Medical Research Council (SAMRC) Immunology of Infectious Diseases, Faculty of Health Sciences, University of Cape Town, Cape Town, 7925 South Africa; 2 International Centre for Genetic Engineering and Biotechnology (ICGEB), Cape Town Component, Cape Town, 7925 South Africa; 3 The Medical Research Centre, Institute of Medical Research and Medicinal Plant Studies, Ministry of Scientific Research and Innovation, Yaoundé, Cameroon; 4 Translational Innovation Platform Immunology, EMD Serono Research and Development Institute, Inc., 45A Middlesex Turnpike, Billerica, MA 01821 USA; 5 The Center for Infectious Disease Research, Seattle, WA 98109 USA; 6 Chemistry Department, Faculty of Science, Cairo University, Cairo, Egypt; 7 Global Health Institute of Merck, Ares Trading S.A. a subsidiary of Merck KGaA Darmstadt Germany, Eysins, Switzerland

**Keywords:** Chronic inflammation, Preclinical research

## Abstract

Tissue fibrosis underlies the majority of human mortality to date with close to half of all reported deaths having a fibrotic etiology. The progression of fibrosis is very complex and reputed irreversible once established. Although some preventive options are being reported, therapeutic options are still scarce and in very high demand, given the rise of diseases linked to fibroproliferative disorders. Our work explored four platforms, complementarily, in order to screen preventive and therapeutic potentials of the antiparasitic drug Praziquantel as a possible antifibrotic. We applied the mouse CCl_4_-driven liver fibrosis model, the mouse chronic schistosomiasis liver fibrosis model, as well as novel 2D and 3D human cell-based co-culture of human hepatocytes, KCs (Kupffer cells), LECs (Liver Endothelial Cells), HSCs (Hepatic Stellate Cells) and/or myofibroblasts to mimic in vivo fibrotic responses and dynamics. Praziquantel showed some effect on fibrosis marker when preventively administered before severe establishment of fibrosis. However, it failed to potently reverse already established fibrosis. Together, we provided a novel sophisticated multi-assay screening platform to test preventive and therapeutic antifibrotic candidates. We further demonstrated a direct preventive potential of Praziquantel against the onset of fibrosis and the confirmation of its lack of therapeutic potential in reversing already established fibrosis.

## Introduction

Discovered close to 50 years ago, Praziquantel (PZQ), a tetracyclic tetrahydroisoquinoline derivative administered as a racemate is the main drug therapy for combating flatworm parasites including tapeworms and schistosomes^1,2^. In vitro and in vivo, the antischistosomal activity of PZQ enantiomers resides primarily in the *(R)-*enantiomer^3,4^. The drug induces rapid paralysis of adult flatworm musculature and tegumental damage that facilitate the immunological removal of the worm from the host^5,6^. As a result, the morbid manifestations of tissue-dwelling flatworm larvae were reported to be considerably, and even at times fully, controlled by PZQ treatment^7–25^. One crippling and life-threatening sequelae resulting from *Schistosoma* eggs trapped in tissue is fibrosis^26,27^. Fibrosis manifests as the uncontrolled formation of extracellular matrix in injured organ that progressively replaces the tissue parenchyma and drives pathology. Reduction of tissue fibrosis following PZQ treatment of flatworm-driven infections and injuries has been reported to be a direct consequence of the antiparasitic effect of the drug^28^. The still poorly defined mode of action of the drug^5,6,29–32^ has made definitive claims on its scope of action difficult. In fact, a recent report claiming PZQ-associated reduction of pathological collagen deposition^14,15,19,21,25,33,34^, has now suggested a potential ability of the drug to also directly treat/reverse established tissue fibrosis.

Hence, we evaluated within this present study whether PZQ may directly affect fibrotic processes when administered preventively and/or therapeutically. We tested the antifibrotic properties of the drug within murine models of acute chemically- or chronic schistosomiasis-induced liver fibrosis. In addition, PZQ was tested in a large panel of human primary cell systems mimicking physiological fibrotic conditions and inflammation. Within these models and assays mimicking establishing or already established fibrosis settings, PZQ and its enantiomers were able to halt the expression of fibrotic factors during the onset of fibrosis but failed to reverse the process once fibrosis had already firmly established.

## Results

### Effects of PZQ treatment in the murine model of carbon tetrachloride (CCl_4_)-induced liver fibrosis

**Figure 1:**
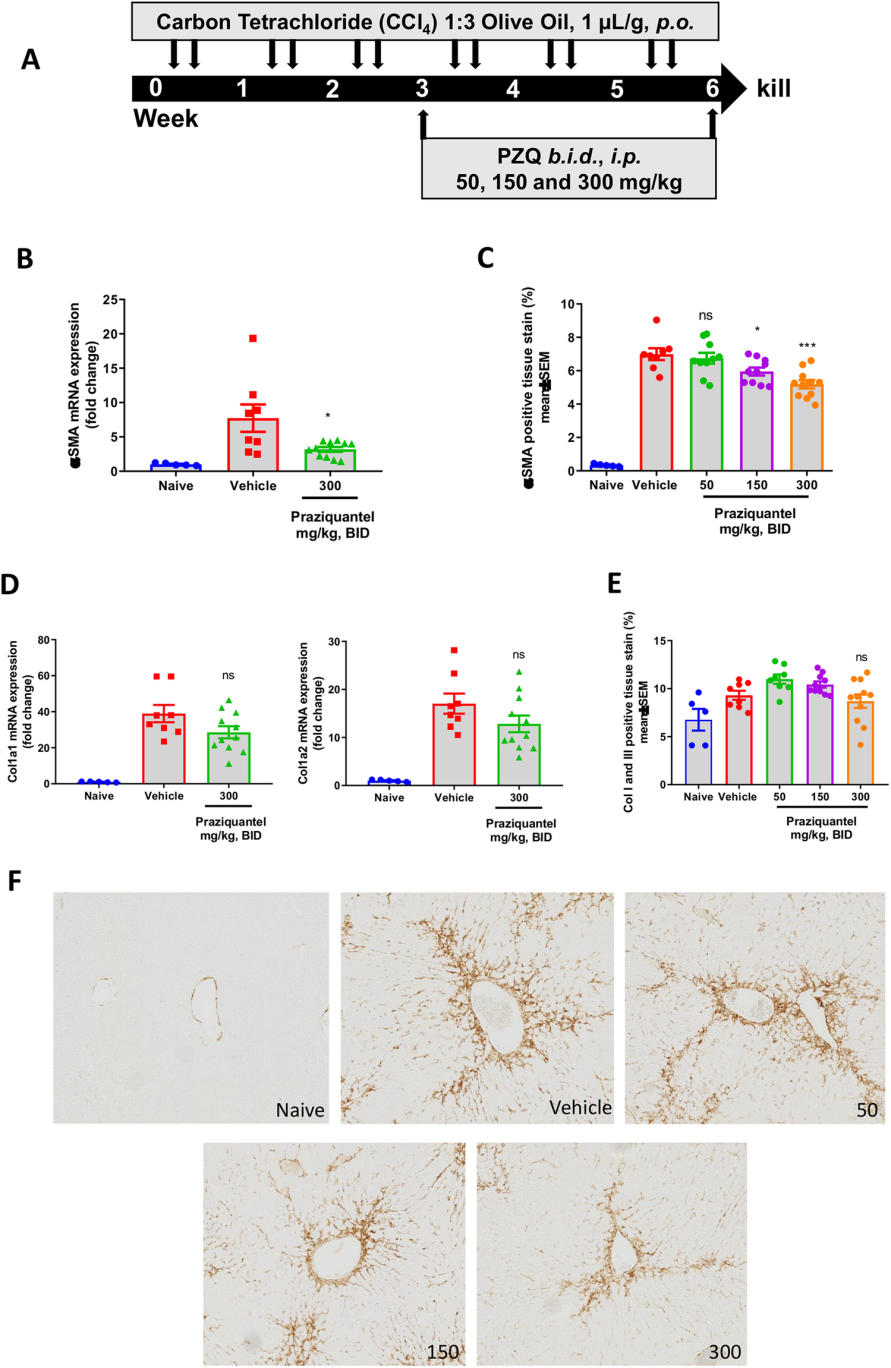
Effect of PZQ on the prevention murine CCl_4_ liver fibrosis model. (**A**) experimental design. Mice were injected twice a week *i.p.* with CCl_4_ in olive oil (1:3) at a vol/weight ratio of 1 µL/g for 6 weeks. Treatment with PZQ or vehicle was initiated 3 weeks after the first CCl_4_ treatment for 3 additional weeks at varying doses of 50, 150 and 300 mg/kg. Animals were euthanized at week 6 post initiation of CCl_4_ treatment and liver samples were collected for histology and qPCR analysis of profibrotic markers (Col1a1, Col1a2, ColII, Col III and αSMA). (**B**) liver *αsma* mRNA levels (Unpaired t-test, *p < 0.0175). (**C**) liver αSMA protein levels around portal vein and perisinusoidal area (1-way ANOVA, ns-not significant; *p < 0.05; ***p < 0.001) (**D**) liver collagen mRNA levels. (Unpaired t-test, ns-not significant) (**E**) liver collagen protein levels, as determined by IHC stain (**F**). (1-way ANOVA, ns-not significant) Results are representative of 2 independent experiments with up to 15 mice per group. Data are presented as means ± SD. *p ≤ 0.05; **p ≤ 0.01; ***p ≤ 0.001.

Initially, we assessed the ability of PZQ to influence liver fibrosis within an in vivo model of chemically-induced hepatic fibrosis. Hence, we used carbon tetrachloride (CCl_4_) and monitored hepatotoxicity and fibrogenesis in mice over a 6-week period (Fig. 1A). For the last three weeks of CCl_4_ treatment, prior to firm establishment of tissue fibrosis, mice were dosed *b.i.d.* for three weeks with PZQ or vehicle (Fig. 1A) with doses of 50, 150 and 300 mg/kg. Animals were then euthanised and serum levels of liver enzymes (AST and ALT) and albumin determined (Figure S1). PZQ treatment failed to alter serum levels of AST (Figure S1A), ALT (Figure S1B) or Albumin (Figure S1C) in CCl_4_-treated mice.

Histological and qPCR analysis of alpha smooth muscle actin (αSMA) and the core fibrosis marker collagen (Fig. 1) in liver lobes was employed to determine the effect of PZQ treatment on liver fibrosis induced by CCl_4_. PZQ, at the highest dose of 300 mg/kg, reduced the expression of αSMA (Fig. 1B,C), but not significantly Col1a1 and Col1a2 mRNAs in the liver, although a trend of collagen expression reduction by increasing doses of PZQ was appreciable (Fig. 1D). In addition, even though the drug failed to significantly reduce tissue collagen deposition, a trend of reduction of collagen fibers in the tissues treated with increasing doses of PZQ was observed (Fig. 1E), as determined by quantification of collagen I/III IHC stain (Fig. 1F). Given the central role of αSMA and deposited collagen in driving tissue fibrosis^35,36^ and the observed dose-dependent reduction of these markers by PZQ treatment, although not significantly, our experiments in this model of acute, CCl_4_-induced liver fibrosis suggest a certain, but minimal, potential of PZQ in limiting the onset of tissue fibrosis when administered twice daily for three weeks prior to establishment of tissue fibrosis in an acute model.

### Effects of PZQ enantiomers in a 3D human microtissue hepatic fibrosis model

**Figure 2:**
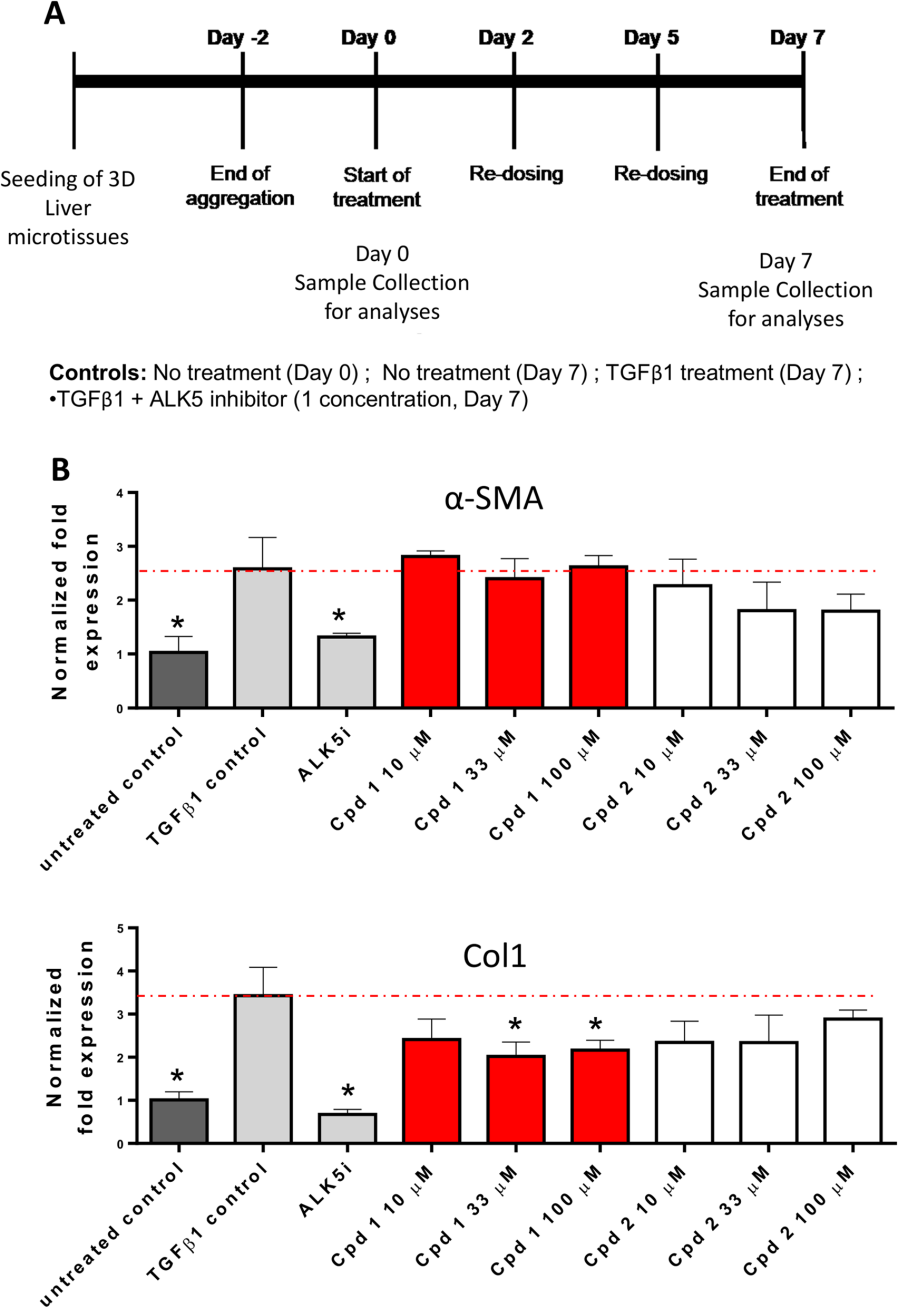
Praziquantel effects in the preventive 3D InSight Human Liver Fibrosis Model. (**A**) Experimental design. Multi-donor hepatocytes were seeded and co-cultivated with Kupffer cells, liver endothelial cells and hepatic stellate cells until aggregation into 3D microtissue for 48 h. Microtissues were then kept in culture for 2 more days and fibrosis was induced by treatment with TGF-beta for 7 days. PZQ enantiomers were added during these 7 days of treatment (at day 2 and day 5) at various concentrations (10, 33 or 100uM). Samples were collected at day 0 and day 7 of treatment for analysis of hepatic stellate cell activation markers gene expression *i.e.* α-SMA and collagen. A control of inhibition was also tested by addition of the TGF-beta specific inhibitor ALK5i. (**B**) Liver microtissues were kept in culture as indicated in (**A**) and kept either without supplements (Untreated control), with TGF-β1 as a positive control of induction of hepatic stellate cell activation (TGF-β1 control), with TGF-β1 and TGF-β1 signaling inhibitor (control to prove efficient inhibition of TGF- β1-induced HSC activation in our system), or cultures supplemented with TGF- β1 and different doses of PZQ enantiomers i.e. (*R)*-PZQ as compound 1 and (L)-PZQ as compound 2 (tests of their ability to inhibit TGF-β1-driven HSC activation). Gene expression relative to the average expression of two housekeeping genes GAPDH and B2M are shown for α-SMA (Upper panel) and Col1 (lower panel) expression. Dashed red lines indicate the expression levels in TGF-beta-only stimulated cultures as reference for statistical analyses. Results are representative of 2 biological replicates. Cpd 1: (*R*)-PZQ, Cpd 2: (*S*)-PZQ. * = p < 0.05.

Next, we further investigated the effect of PZQ treatment (using either R or S enantiomers) on multi-donor hepatocytes seeded and co-cultivated with Kupffer cells, liver endothelial cells and hepatic stellate cells until aggregation into 3D microtissue for 48h^37,38^. Microtissues kept in culture were treated with TGF-β for 7 days and PZQ enantiomers were added during these 7 days of treatment (at day 2 and day 5) at various concentrations (10, 33 or 100 µM, as shown in Fig. 2A). None of the tested doses of the drugs elicited cytotoxicity, as measured by ATP release in our assay (Figure S2). Gene expressions of αSMA and collagen relative to the housekeeping gene GAPDH or B2M revealed overall a dose-dependent ability of PZQ enantiomers to reduce the expression of fibrosis markers (Fig. 2B), when administered during the course of the TGF-β-driven induction of fibrosis in hepatic stellate cells-containing 3D microtissues. A case is therefore made here for the potential of PZQ and its enantiomers to oppose the onset of fibrosis.

### Effects of PZQ in the murine model of established liver fibrosis following chronic experimental schistosomiasis

**Figure 3:**
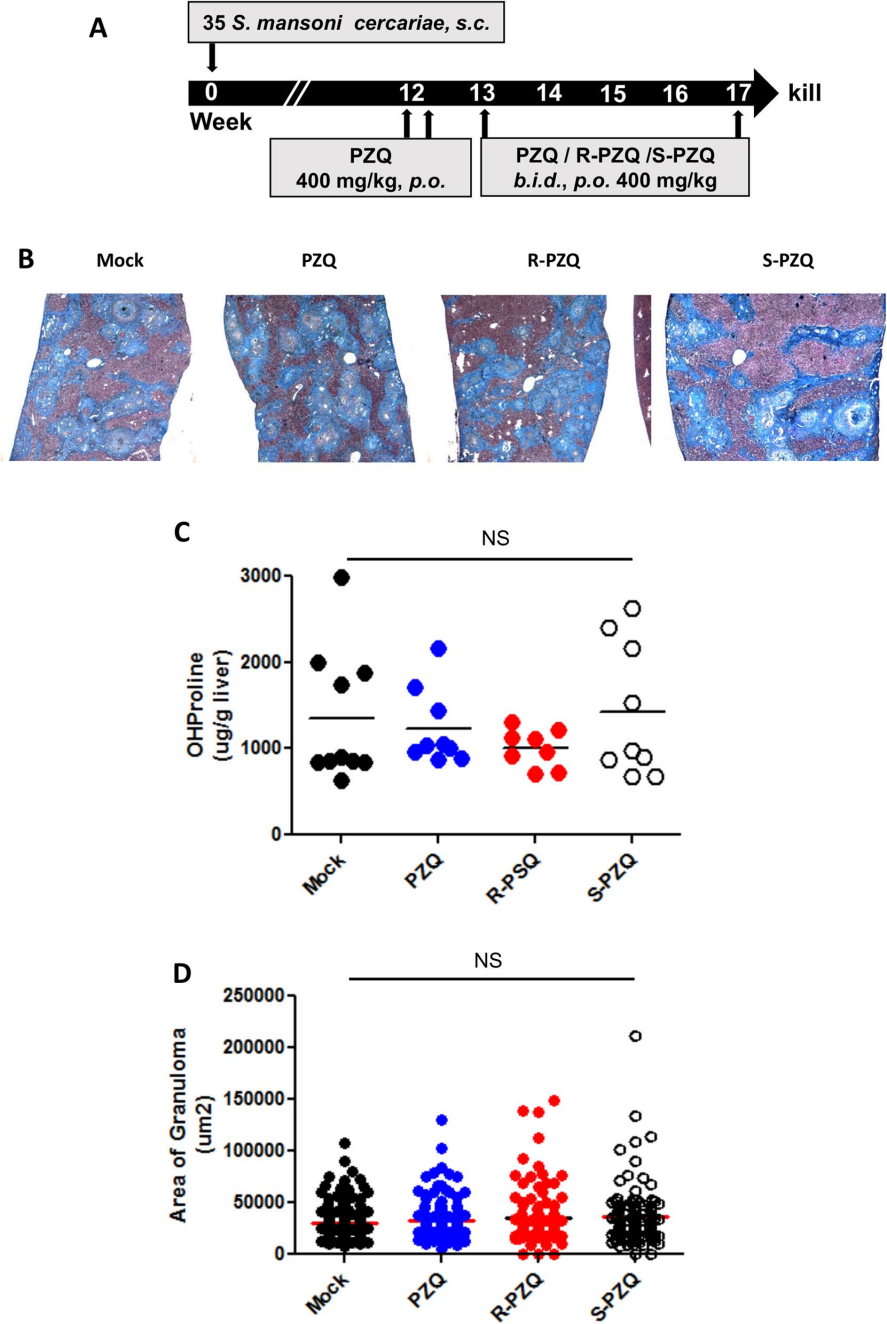
Praziquantel effects in the chronic murine schistosomiasis model of liver fibrosis. (**A**) Experimental design. Mice were infected percutaneously with 35 cercariae of *S. mansoni* and treated twice with PZQ (400 mg/kg) at week 12 post infection to clear the worms. Following anti-parasitic treatment, mice were further treated with racemic PZQ or its enantiomers (400 mg/kg) twice daily for 4 weeks and killed thereafter for analysis of liver fibrosis features. (**B**) CAB staining of liver sections depicting collagen deposition in blue. **C.** Hydroxyproline measurement in the liver biopsies of PZQ-treated mice (Unpaired t-test, ns-not significant). **D.** Granuloma areas surrounding trapped eggs in the liver of PZQ-treated mice (Unpaired t-test, ns-not significant). Results are representative of 2 independent experiments with 7–10 mice per group.

We further investigated the in vivo effect of PZQ treatment on clinical liver fibrosis by employing a murine model of chronic experimental schistosomiasis. C57BL/6 mice were infected percutaneously with a low dose of *Schistosoma mansoni* cercariae (35 per animal). After 12 weeks of infection, mice were treated *p.o.* twice with 400 mg/kg of PZQ over a week period to clear all adult worms and halt egg production. Following this first step of antiparasitic treatment with PZQ, animals were further treated with *p.o.* doses of 400 mg/kg PZQ or its enantiomers (*R* or *S*) administered *b.i.d.* for 4 weeks (Fig. 3A). Antifibrotic treatment of deparasitized animals with PZQ or its enantiomers failed to result in any detectable amelioration of the liver fibrotic profiles. This is shown by CAB staining (Fig. 3B), liver hydroxyproline levels (Fig. 3C), as well as egg-driven granulomatous inflammation (Fig. 3D), measured by the area of the perioval granulomas in the liver of treated animals.

The second round of treatment aimed to uncover a possible direct and therapeutic, rather than preventive, antifibrotic potential of PZQ, which might be independent from its known antiparasitic effect. This round of treatment did not result in an additional reduction of liver egg burden in animals confirming the efficiency of the first round of antiparasitic treatment in clearing all subsequent egg production in the treated animals (Figure S3A).

These results suggest efficient clearing of all egg-producing *S. mansoni* adult worms by the first antiparasitic regimen in *S. mansoni*-infected mice. Indeed, no reduction in the liver egg burden could be observed following a subsequent antifibrotic treatment of 4 weeks with PZQ when compared to animals receiving a mock treatment of 4 weeks instead. When comparing mock-treated deparasitzed animals with PZQ-treated counterparts, we also noted no alteration of serum levels of liver enzyme ratio after such an extended PZQ treatment (Figure S3B). This supports the safe nature of the drug and its enantiomers in our present assay. Taken together, our data show that prolonged and repeated treatments with PZQ or its enantiomers at high doses did not directly affect established liver fibrosis in the context of chronic murine schistosomiasis.

### Phenotypic profiling of PZQ in Human primary cell systems panel

**Figure 4:**
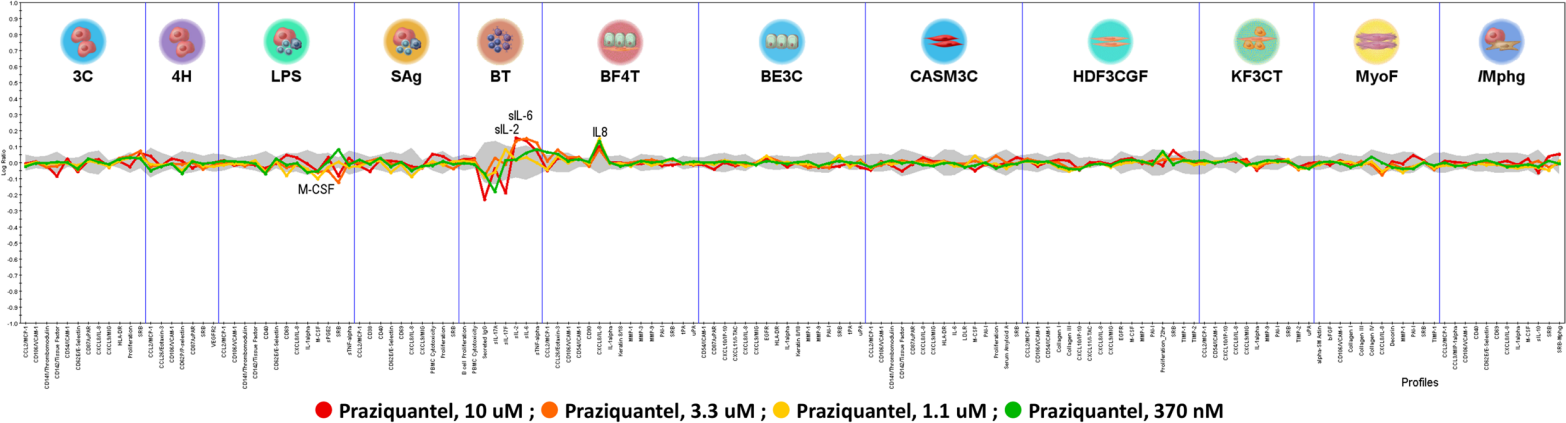
Praziquantel effects in the BioMAP diversity panel. Profile of PZQ effects on the Diversity PLUS Panel. Human primary cells in the systems are used at early passage (4 or earlier) to minimize adaptation to cell culture conditions and preserve physiological signalling responses. The x-axis lists the quantitative protein-based biomarker readouts measured in each system. The Y-axis represents a log-transformed ratio of the biomarker readouts for the PZQ-treated sample over vehicle controls. The grey region around the Y-axis represents the 95% significance envelope generated from historical vehicle controls. Biomarker activities are annotated when 2 or more consecutive concentrations change in the same direction relative to vehicle controls, are outside of the significance envelope, and have at least one concentration with an effect size > 20% (|log1- ratio| > 0.1). Biomarker key activities are described as modulated if these activities increase in some systems, but decrease in others. Cytotoxicitiy is indicated on the profile plot by a thin black arrow above the X-axis, and antiproliferative effects are indicated by a thick grey arrow. All cells are from a pool of multiple donors (n = 2–6), commercially purchased and handled according to the recommendations of the manufacturers. X-axis from left to right: CCL2/MCP−1; CD106/VCAM−1; CD141/Thrombomodulin; CD142/Tissue Factor; CD54/ICAM−1; CD62E/E−Selectin;CD87/uPAR; CXCL8/IL−8; CXCL9/MIG; HLA−DR; Proliferation; SRB; CCL2/MCP−1; CCL26/Eotaxin−3; CD106/VCAM−1; CD62P/P−selectin; CD87/uPAR; SRB; VEGFR2; CCL2/MCP−1; CD106/VCAM−1; CD141/Thrombomodulin; CD142/Tissue Factor; CD40; CD62E/E−Selectin; CD69; CXCL8/IL−8; IL−1alpha; M−CSF; sPGE2; SRB; sTNF−alpha; CCL2/MCP−1; CD38; CD40; CD62E/E−Selectin; CD69; CXCL8/IL−8; CXCL9/MIG; PBMC Cytotoxicity; Proliferation; SRB; B cell Proliferation; PBMC Cytotoxicity; Secreted IgG; sIL−17A; sIL−17F; sIL−2; sIL−6; sTNF−alpha; CCL2/MCP−1; CCL26/Eotaxin−3; CD106/VCAM−1; CD54/ICAM−1; CD90; CXCL8/IL−8; IL−1alpha; Keratin 8/18; MMP−1; MMP−3; MMP−9; PAI−I; SRB; tPA; uPA; CD54/ICAM−1; CD87/uPAR; CXCL10/IP−10; CXCL11/I−TAC; CXCL8/IL−8; CXCL9/MIG; EGFR; HLA−DR; IL−1alpha; Keratin 8/18; MMP−1; MMP−9; PAI−I; SRB; tPA; uPA; CCL2/MCP−1; CD106/VCAM−1; CD141/Thrombomodulin; CD142/Tissue Factor; CD87/uPAR; CXCL8/IL−8; CXCL9/MIG; HLA−DR; IL−6; LDLR; M−CSF; PAI−I; Proliferation; Serum Amyloid A; SRB; CCL2/MCP−1; CD106/VCAM−1; CD54/ICAM−1; Collagen I; Collagen III; CXCL10/IP−10; CXCL11/I−TAC; CXCL8/IL−8; CXCL9/MIG; EGFR; M−CSF; MMP−1; PAI−I; Proliferation_72hr; SRB; TIMP−1; TIMP−2; CCL2/MCP−1; CD54/ICAM−1; CXCL10/IP−10; CXCL8/IL−8; CXCL9/MIG; IL−1alpha; MMP−9; PAI−I; SRB; TIMP−2; uPA; alpha−SM ; Actin; bFGF; CD106/VCAM−1; Collagen I; Collagen III; Collagen IV; CXCL8/IL−8; Decorin; MMP−1; PAI−I; SRB; TIMP−1; CCL2/MCP−1; CCL3/MIP−1alpha; CD106/VCAM−1; CD40; CD62E/E−Selectin; CD69; CXCL8/IL−8; IL−1alpha; M−CSF; sIL−10; SRB; SRB−Mphg.

We then examined the effects of PZQ in twelve 2D primary human cell culture systems provided by BioMAP simulating multiple diseased tissues. The effect of PZQ against relevant pathways as well as cell interactions was measured through biomarker readouts, such as proteins and small molecule mediators in the BioMAP panel of systems^39^. Here, PZQ was tested in the BioMAP panel at several doses (0.37, 1.1, 3.3 and 10 µM). We observed its ability to downregulate expression of M-CSF in the cellular system of monocyte-induced Th1 activation (Fig. 4). Conversely, PZQ distinctively prompted the upregulation of sIL-2 and sIL-6 in the T-cell-dependent B cell activation system and the increase of IL-8 in the Th2 lung airway inflammation system. However, PZQ completely failed to repress any of the established fibrotic cell systems or associated biomarkers in our screened BioMAP panel of systems at all tested concentrations. This includes endothelial cell proliferation, angiogenesis, Th2 vascular inflammation, expression of matrix metalloproteases MMP-1, MMP-3 or MMP-9, expression of tissue inhibitors of matrix metalloproteases TIMP-1 or TIMP-2 and importantly expression of collagen I, collagen III or collagen IV. Taken together, in the human primary cell-based systems tested, where the fibrosis process is already established, PZQ had no effect, at the tested doses, to reverse an already established fibrosis process.

## Discussion

Praziquantel is the drug of choice to combat flatworm infections. The extensive usage of PZQ during mass drug administration campaigns in flatworm-endemic areas has considerable health benefits for the target populations over time such as improved fitness, improved cognitive functions and reduced tissue fibrosis^13,15–20,34,40–52^. Whereas most authors associated such health benefits and particularly the improvement of tissue fibrosis in helminth-infested individuals to the sole antiparasitic potential of PZQ^9,11,21,53–63^, recent reports on the drug ability to also directly mitigate tissue fibrosis have also emerged^13,15–20,34,40–52^ presenting PZQ as a potential new antifibrotic.

The direct antifibrotic potential of PZQ was first investigated in a standard in vivo mouse model of CCl_4_-induced liver fibrosis^35^. The potential influence of PZQ during the onset of fibrotic processes in vivo where all intercellular interactions are present was studied. Although twice daily dosing was given over several weeks, PZQ modestly reduced the expression of αSMA, a marker for activation of stellate cells and showed minimal but non-significant ability to reduce the expression or deposition of the main fibrotic matrix protein collagen in the CCl_4_-induced fibrotic livers. This data suggests that PZQ has very limited but apparent antifibrotic activities in the model. This is in agreement with recent reports of PZQ ability to oppose fibrosis onset in a murine model of acute fibrosis^33^.

PZQ enantiomers were then administered concomitantly with pro-fibrogenic TGF-β1 in the in vitro InSight Human Liver Fibrosis Model 3D system, a model where fibrosis onset parallels the addition of the drug for assessment of preventive potential, similarly to the CCl_4_-based model. Here also, we observed a minimal but dose-dependent ability of the drug to downregulate expression of αSMA and collagen. Clearly, in models of acute fibrosis, PZQ and its enantiomers appear to possess a minimal but perceptible antifibrotic potential. Although of value, the administration of PZQ only speaks to the prevention of fibrosis progression in clinical settings. The general situation remains that of attempts to reverse already established fibroproliferative diseases, due to their poorly symptomatic natures in affected patients. We therefore questioned the therapeutic potential of PZQ and its enantiomers in their ability to reverse already established tissue fibrosis. To address this, we used the more robust and extended model of fibrosis elicited in the liver of mice chronically affected by schistosomiasis^55,64^. We tested how prolonged PZQ (racemate and enantiomers) treatment (400 mg/kg, twice-daily for 4 weeks) would perform on already established liver fibrosis in chronic schistosomiasis, where trapped parasite eggs cause the tissue fibrosis even after removal of parasite worms following deparasitization. We observed that PZQ or its enantiomers were unable to reduce the levels of liver fibrosis in PZQ-treated animals after such an extended and pronounced scheme. Therefore we conclusively argued for the inability of PZQ to efficiently reverse already established and severe tissue fibrosis. In fact, reversal of liver fibrosis and digestion of deposited collagen is a time-consuming process requiring several weeks post treatment to become apparent^65^. Using another model of established fibrosis, PZQ, tested in the DiscoverX BioMAP panels, modulated the expression of several cytokines but did not affect the expression of markers of myofibroblast activation and fibrosis related matrix activities such as αSMA and collagens. This suggests that PZQ was active at the dose range tested in such in vitro assays but might simply not be able to reverse an already firmly established fibrotic process as opposed to the two approved antifibrotic agents pirfenidone and nintedanib, which reportedly exhibited antifibrotic effects in this system^66^. Taken together, this supports the observation that, in our settings, PZQ and its enantiomers did not succeed in therapeutically opposing the persistence of already established fibrotic tissue.

Here, we provide an experimentally well-balanced basis for our conclusion of potential preventive potential of PZQ against acute fibrosis and a lack of a direct antifibrotic potential in reversing established chronic fibrosis. Our findings align with the systematic appraisal of the literature by the group of Andrade^28^ indicating that PZQ suggested antifibrotic potential in chronic schistosomiasis patients is essentially dependent on its antiparasitic effect. In fact, it is widely observed that halting injury or stopping the injury-inducing parasite should considerably help reduce the fibrotic pathology over time^1,7,9,15,20,21,34,65^. Additionally, worm killing by PZQ at a relatively late stage leads to less fibrosis in schistosomiasis-diseased animals over time when compared to untreated infected animals^67^. This might therefore explain that the recent study associating PZQ with a direct antifibrotic effect in particular^47^ and an anti-pathological effect in general, might only hold true in the context of preventive treatment schemes. Therefore, the more recent observation of an antifibrotic effect of PZQ when administered preventively in the non-infectious CCl_4_ liver fibrosis model^33^ argue in favour of this nuanced ability of PZQ to prevent the onset of fibrosis while failing to demonstrate any therapeutic potential of the drug on already established tissue fibrosis. Our present study now reconcile such observations, demonstrating that PZQ has potential preventive antifibrotic effects while it has no efficacy in therapeutically reversing established fibrosis. This conclusion reliably recapitulates the established view of most authors on the subject^9,11,21,53–63^ where PZQ antifibrotic / anti-pathological potential in clinically fibrotic schistosomiasis-diseased patients might not be direct and/or effective on established fibrosis but entirely consequent to its anti-parasitic effect when administered after fibrosis development.

In summary, we show that PZQ does not counter established fibrotic processes nor reduce pre-existing tissue fibrosis independently from its known antiparasitic effect in schistosomiasis but harbours a potential in preventing the onset of the fibrotic process before it is established. This has great implications for the mass administration of this drug in sub-Saharan countries as it speaks to the potential additional but poorly addressed benefit of the drug as an unintended antifibrotic agent. Caution is nevertheless raised on the timely and now better informed use of PZQ for prevention, rather than a therapeutic candidate against tissue fibrosis.

## Materials and methods

### Phenotypical profiling on CCl_4_-based model of murine liver fibrosis

#### Mice and ethics

The use of animals was approved by the University of Cape Town Animal Ethics Committee (Protocol 016/027) and all animal experiments were performed in accordance with the South African National Standard (SANS 10386:2008) and in accordance with the EMD Serono Institutional Animal Care and Use Committee (IACUC). All mice were fed standard chow ad libitum and exposed to standard light:dark sleep cycles.

#### Murine CCl_4_ fibrosis model

Male and female Balb/C mice (9–11 week old) (Jackson Labs) were randomized in a blinded fashion and then assigned to treatment groups. To induce liver fibrosis, mice were injected twice weekly starting from day 0 with a 1:3 solution of carbon tetrachloride (CCl_4_) (Sigma) in olive oil (Sigma) for a total of 6 weeks. Mice were therapeutically treated by oral gavage (*p,o.*), twice daily (BID) for the last 3 weeks of the study. At 6 weeks mice were euthanized by CO_2_ asphyxiation, sera and liver were collected for analysis.

#### Preparation of solutions (PZQ and CCl_4_) and administration to mice

CCl_4_ (Sigma) was diluted in olive oil (Sigma) in a 1:3 ratio. The final preparation was injected in each mouse intraperitoneally (*i.p.*) at 1 µL/g with a glass syringe (Hamilton) and a 27 g × ½ needle (Becton Dickenson) except for sham. Carbon tetrachloride in olive oil was prepared fresh for each injection.

PZQ (EMD Serono) for oral administration was prepared at 50, 150 and 300 mg/kg in 10% Kolliphor HS15 (Sigma) in water. Drug suspensions were mildly stirred during collection of drug into the dosing syringe prior to oral gavage.

#### Sampling (serum, liver)

After CO_2_ asphyxiation, 500–600 µL of whole blood was collected by cardiac puncture into a serum microcollection tube (Becton Dickenson). After coagulation, whole blood was centrifuged at 10,000 rpm for 10 min, 4 °C. Serum was collected and frozen at -20 °C for serum liver enzyme and albumin analysis. The medial and left lobe was collected, split in thirds and flash frozen for qPCR. The left lobe was preserved in 10% neutral buffered formalin for immunohistochemistry and general histology.

#### Quantification of liver enzymes and albumin in sera

Serum samples were thawed and kept on ice until analysis. AST, ALT, and albumin were measured using the Siemens Advia 1800 clinical chemistry machine according to manufacturer’s instructions. Serum samples were run undiluted and then diluted with distilled water as necessary to obtain values in range of assay. Liver enzymes are reported as (U/L) and albumin is reported as (g/dL).

#### RNA extraction and cDNA synthesis and qPCR from liver tissue

Total RNA was extracted from the liver homogenate using an RNeasy kit (Qiagen). Complementary DNA was synthesized from total liver RNA (800 ng) using Superscript VILO cDNA synthesis kit (Invitrogen) under manufacturer’s instructions with Thermacycler (BioRad) incubation conditions as follows, 25 °C for 10 min, 42 °C for 60 min, and then 85 °C for 5 min with 4 °C infinite hold. Final concentration of cDNA was brought to 5 ng/µL with RNase-free water. Quantitative RT-PCR was performed according to instructions provided in the Taqman n Gene expression kit (Invitrogen). All samples were run in duplicate. Taqman primers measured were Col1a1 (Mm00801666_g1), Col1a2 (Mm00483888_m1), Acta1 (Mm00808218_g1), GADPH (Mm99999915_g1). The amplification reaction was carried out using Quantstudio 12 K flex (Applied Biosystems) under standard Taqman plate conditions. The levels of target gene mRNAs were normalized against the level of GAPDH mRNA via the 2-ΔΔCt method.

#### Liver histology and quantification

Upper, middle, and lower transectional pieces of the left lobe were stained for total collagen by Immunohistochemistry stains including alpha smooth muscle actin *i.e.* αSMA (Abcam, cat# ab124964, 1:2000), Collagen I/III (I: Boster Bio, cat# PB9939, 1:250; III: Abcam, cat# ab7778, 1:100), were performed using the Leica Bond RX. Primary antibodies were labelled using the Agilent Envision + Rabbit Horseradish peroxidase (HRP) kit (cat# K4011) which includes the secondary HRP labelled antibodies allowing for 3,3′-diaminobenzidine (DAB) development. Slides were digitally scanned using the Hamamatsu Nanozoomer Scanner and Digital Pathology Software. Saved images were reviewed and reduced to 1.5% zoom using Hamamatsu NDP view software. Final images were analyzed by threshold analysis of positively stained cells using Image Pro Premier. Final values were reported as percent of positively stained pixels of total measured area.

#### Statistical analysis

All data were graphed and analyzed using Graphpad Prism 7. 1-way ANOVA was used to assess the effects of PZQ compared to vehicle control.

### Phenotypical profiling on a 3D InSight human liver fibrosis model

#### Preparation of PZQ solution

PZQ enantiomers provided as solid powder by Merck KGaA (Darmstadt Germany) were dissolved in DMSO to reach a concentration of 20 mM. Serial dilutions in water were made to test reach concentrations of 10, 33, 100 µM. Control consisted of water and 0.5% DMSO.

#### 3D InSight model profiling

Fibrosis is induced by 7-day exposure to TGF-β on spheroids or 3D Insight Human Liver microtissues comprised of primary human hepatocytes, HSCs, Kupffer cells, and LECs^37,38^. 3D InSight Human Liver Microtissues were provided by InSphero (InSphero AG, Schlieren, Switzerland), in an Akura 96 plate. Microtissues were generated by self-assembly of monodispersed primary cells, as described previously1-3. The liver microtissues were a co-culture of primary human hepatocytes (10-donor pool), primary human non-parenchymal cells (single-donor liver endothelial cells and Kupffer cells) and human primary hepatic stellate cells (single-donor). Following self-assembly, liver microtissues were cultures for 1–4 days in 70 µL of 3D InSight Human Liver Maintenance Media—TOX (InSphero AG) to stabilize phenotypic and functional characteristics. Fibrosis-induced control microtissues were further exposed for 7 days to TGFβ1 and anti-fibrotic control was generated by co-treatment of microtissues with TGFβ1 and ALK5 inhibitor (Alk5i). To assess compound efficacy, liver microtissues were exposed to TGFβ1 and test compounds. Medium exchanged followed by re-dosing was on days 0, 2, and 5 of treatment. The fibrotic disease state is detected by increased expression of hepatic stellate cells activation markers (i.e. α-SMA and collagen).

### Phenotypical profiling on the chronic schistosomiasis model of liver fibrosis

#### Mice and ethics

The use of animals was approved by the University of Cape Town Animal Ethics Committee (Protocol 016/027) and all animal experiments were performed as previously described^68^.

#### Schistosoma mansoni infection

As previously reported^68^, *Biomphalaria glabrata* snails (a gift from Adrian Mountford, York, UK) and NMRI female mice were used to maintain and expand the *S. mansoni* parasite larvae. Anaesthetised mice were percutaneously infected through the abdomen for 30 min with a dose of 35 live *S. mansoni* cercariae distributed by BEI Resources, (NIAID, NIH, USA), *passaged in Biomphalaria glabrata*, through infection of NMRI mice, strain NR-21962.

#### Preparation of PZQ solution and administration schemes

For the oral application, PZQ (Merck) was weighted at quantities sufficient for 400 mg/kg of animal body weight into suitable graduated and labelled containers. 10 parts 70% Tween/30% Ethanol (EtOH) were added and mixed using a magnetic stirrer. Next, 90 parts of distilled sterile water were slowly added. Stirring was continued until a homogeneous suspension was obtained. Formulations were kept under magnetic stirring until the end of each treatment. The suspensions were administered within 3 h after preparation. The volume of administration to mice should be close to 200 µL. *S. mansoni*-infected mice were administered by oral gavage twice weekly 12 weeks post infection to deparasitize. To further assess the direct effect of PZQ on the remaining tissue fibrosis, animals were treated after deparasitation twice daily with 400 mg/kg of PZQ for 4 weeks. As a Mock control group, deparasitized animals were rather treated with a similar volume of carrier solution only i.e. 10 parts 70% Tween/30% EtOH were added and mixed using a magnetic stirrer with 90 parts of distilled sterile water.

#### Sampling (Liver, serum)

*S. mansoni*-infected and deparasitized animals were euthanised after 4 weeks of PZQ treatment and exsanguinated. Blood was collected and centrifuged in serum separator tubes (BD Bioscience, San Diego, CA) at 8000×g for 10 min at 4 °C to retrieve the serum. The upper aqueous serum phase was aliquoted into tubes and stored at -80 °C until further use. Livers were excised then approximately one-third of each liver was stored in formalin for histology, a third used for egg count and the last third used to estimate collagen as hydroxyproline as previously described^69^.

#### Measurement of liver enzymes in serum

Serum levels of alanine and aspartate transaminases were measured at the National Health Laboratory Service of South Africa (Cape Town) to assess the level of hepatic damage in the experimental animals.

#### Liver egg burden determination

Approximately one-third of the liver was used to count schistosome eggs after digestion in 4% KOH_aq_ for 18 h. Liver sections from infected animals and eggs were purified then counted as previously described^69^.

#### Liver histology, granuloma size measurement and fibrosis quantification.

Liver samples were fixed in neutral buffered formalin, processed, and 5–7 μm sections stained with hematoxylin and eosin (H & E), as previously described^68^. The diameters of up to 50 egg-induced granulomas were determined per animal on an ocular micrometer (Nikon NIS-Elements, Nikon Corporation, Tokyo, Japan). Liver sections were then stained with chromotrope 2R and analine blue solution (CAB) and counterstained with Wegert's hematoxylin for collagen staining to appraise the level of fibrosis in the tissues, as previously defined^68,69^. To further quantify collagen deposition, thus fibrosis in the liver tissues, tissue hydroxyproline was quantified, as described 59.

#### Statistical analysis

GraphPad Prism 6 software (https://www.prism-software.com) was used for analyses. Values were displayed as mean ± SD and differences between groups were tested for significance by unpaired Student's t test. Values of p < 0.05 were considered statistically significant.

### Phenotypical profiling on a human primary cell systems panel

#### Preparation of PZQ solution

A 10 mM DMSO solution of praziquantel was shipped at 4 °C to Eurofins, and subsequently diluted with H_2_O to be tested at 10, 3.3, 1.1, and 0.3 µM in the BioMAP Diversity PLUS Panel.

#### BioMAP profiling

A 10 mM DMSO solution of PZQ was shipped at 4 °C to Eurofins to determine the profile of the drug in more complex primary human cell systems. We had the compound tested in the BioMAP Diversity PLUS Panel (BioSeek LLC, South San Francisco, CA) at various dilutions i.e. 10, 3.3, 1.1, and 0.3 µM. The compound was tested across 12 BioMAP Systems. These systems contain primary human cells stimulated with different stimuli. These systems have been described previously^39^ A BioMAP activity profile was generated after addition of PZQ to the stimulated primary human cultures. This profile summarizes the effect(s) of PZQ, tested here, on readouts such as cytokines or growth factors, surface and cellular proliferation. For more technical details, see^39^.

## Supplementary information

Supplementary information

## Data Availability

All reported and supplementary data are available and included in the present manuscript.
