# Supplementary material for: Investigating the antifibrotic effect of the antiparasitic drug Praziquantel in in vitro and in vivo preclinical models
Source: Sci Rep. 2020 Jun 30;10:10638. doi: 10.1038/s41598-020-67514-4 (PMC7327036; doi:10.1038/s41598-020-67514-4)
Supplement: Supplementary file 1 — Supplementary information [file 41598_2020_67514_MOESM1_ESM.pdf]

Investigating the antifibrotic effect of the antiparasitic drug Praziquantel in *in vitro* and *in vivo* preclinical models

Justin Komguep Nono[1,2,3], Kai Fu[4], Thabo Mpotje[1,2], Georgianna Varrone[4], Nada Abdel Aziz[1,2,5], Paballo Mosala[1,2], Lerato Hlaka[1,2] , Severin Donald Kamdem[1,2] , Daigen Xu[4], Thomas Spangenberg[6#], Frank Brombacher[1,2#]

[1]University of Cape Town, Division of Immunology and South African Medical Research Council (SAMRC) Immunology of Infectious Diseases, Faculty of Health Sciences, University of Cape Town, Cape Town 7925, South Africa.

[2]International Centre for Genetic Engineering and Biotechnology (ICGEB), Cape Town Component, Cape Town 7925, South Africa.

[3]The Medical Research Centre, Institute of Medical Research and Medicinal Plant Studies, Ministry of Scientific Research and Innovation, Yaoundé, Cameroon.

[4]Translational Innovation Platform Immunology, EMD Serono Research and Development Institute, Inc., 45A Middlesex Turnpike, Billerica, MA 01821, and The Center for Infectious Disease Research, Seattle 98109, WA, USA.

[5]Chemistry Department, Faculty of Science, Cairo University, Cairo, Egypt.

[6]Global Health Institute of Merck, Ares Trading S.A., a subsidiary of Merck KGaA Darmstadt Germany, Eysins, Switzerland.

SUPPLEMENTARY FIGURES AND LEGENDS

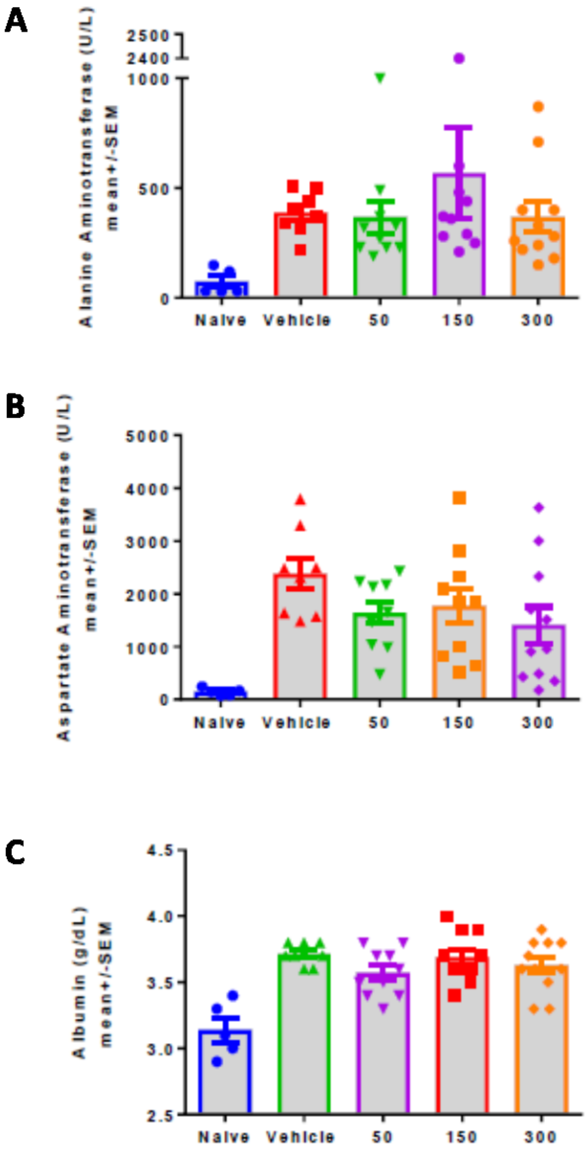

Figure S1: Liver enzymes and albumin levels in the serum of CCl_4-treated mice. **A.** ALAT levels in PZQ-treated animals in the Ccl4 model. **B.** ASAT levels in PZQ-treated animals in the Ccl4 model, **C.** Albumin levels in PZQ-treated animals in the Ccl4 model. (1-way ANOVA, ns-not significant). Results are representative of 2 independent experiments with up to 15 mice per group.

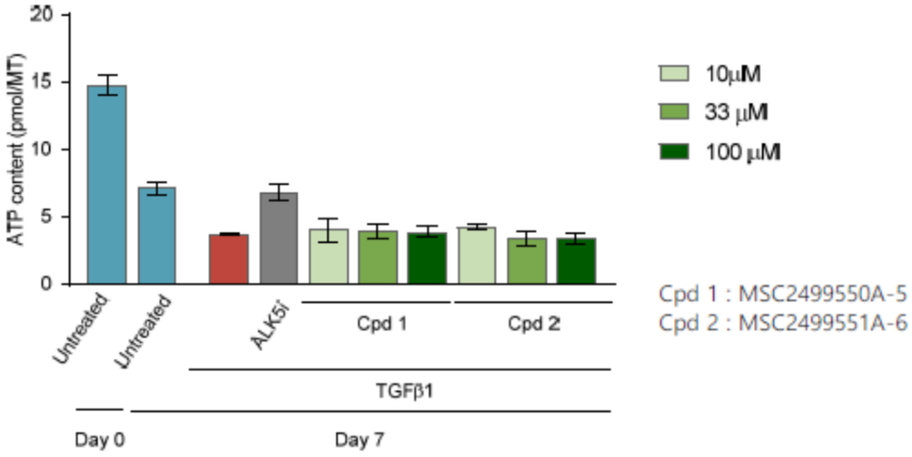

Figure S2: The effect of indicated treatments with PZQ enantiomers on cell viability in the 3D microtissue model. ATP content was measured as an indicator of cell viability for different stimulation conditions for 3D human microtissue cultures. Cpd 1: (*R*)-PZQ, Cpd 2: (*S*)-PZQ.

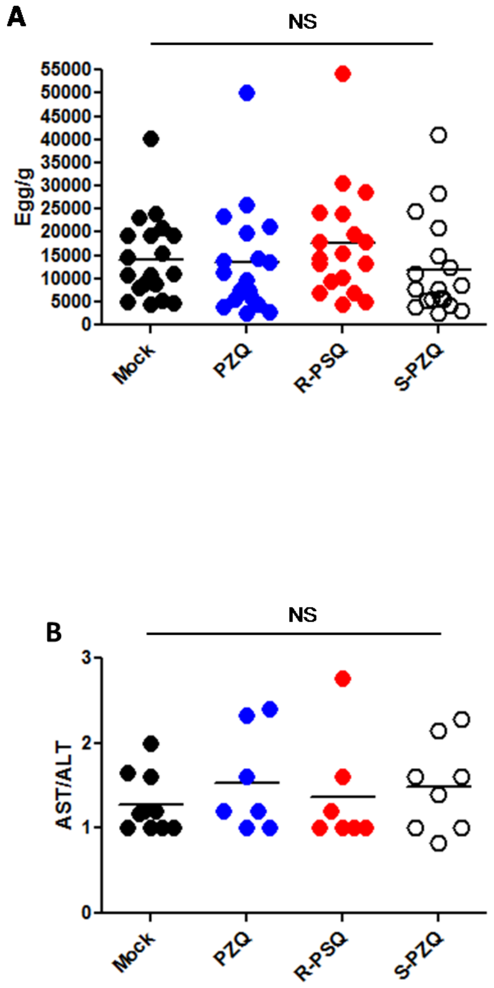

Figure S3: Egg burdens and liver enzymes in *S. mansoni*-infected then PZQ-treated mice. A. Egg burdens. (Unpaired t-test, ns-not significant). **B.** AST/ALT ratio in the serum of mice (Unpaired t-test, ns-not significant). Results are representative of 2 independent experiments with 10 mice per group
